# Health Conditions in Adults With Cerebral Palsy: The Association With CP Subtype and Severity of Impairments

**DOI:** 10.3389/fneur.2021.732939

**Published:** 2021-10-28

**Authors:** Ulrica Jonsson, Meta N. Eek, Katharina Stibrant Sunnerhagen, Kate Himmelmann

**Affiliations:** ^1^Department of Clinical Neuroscience, Institute of Neuroscience and Physiology, Sahlgrenska Academy, University of Gothenburg, Gothenburg, Sweden; ^2^Region Västra Götaland, Habilitation and Health, Adult Habilitation, Gothenburg, Sweden; ^3^Department of Health and Rehabilitation, Institute of Neuroscience and Physiology, Sahlgrenska Academy, University of Gothenburg, Gothenburg, Sweden; ^4^Department of Pediatrics, Institute of Clinical Sciences, Sahlgrenska Academy, University of Gothenburg, Gothenburg, Sweden

**Keywords:** cerebral palsy, health conditions, CP subtype, intellectual disability, prevalence, gross motor function classification system, comorbidities

## Abstract

**Aim:** To determine the prevalence of common health conditions in adults with cerebral palsy (CP) and to identify associations with the CP subtype or the severity of impairments.

**Methods:** A population-based, cross-sectional study of 153 adults with CP born from 1959 to 1978 (87 males, 66 females; median age 48 years 3 months, range 37–58 years; 41% with unilateral spastic, 36% bilateral spastic, 19% dyskinetic, and 4% with ataxic CP). Data was gathered through interviews, physical assessments, and medical record reviews.

**Results:** The most common health conditions in adults with CP were pain 65%, upper gastrointestinal disorders 33%, dysphagia 29%, epilepsy 29%, and depression 27%. Cerebral palsy subtype was significantly associated with the presence of pain (*p* = 0.029), gastrointestinal (*p* < 0.001), and respiratory disorders (*p* = 0.006). A more severe physical impairment was associated with a higher prevalence of gastrointestinal disorders (*p* < 0.001), respiratory disorders (*p* < 0.001), and pressure ulcers (*p* < 0.001). Intellectual disability was associated with a higher prevalence of gastrointestinal disorders (*p* < 0.001), pneumonia (*p* = 0.001) epilepsy (*p* = 0.001), and pressure ulcers (*p* < 0.001), but with a lower prevalence of pain (*p* < 0.004) and hypertension (*p* = 0.043).

**Conclusion:** The prevalence of several common health conditions is related to the CP subtype and severity of impairments, indicating that CP plays a role in the development of these health conditions. Follow-up of adults with CP needs to include not only impairments, but general health as well. Increased attention directed toward signs of gastrointestinal and respiratory disorders in individuals with either dyskinetic CP, gross motor function classification system (GMFCS) levels IV–V, or intellectual disability, is recommended.

## Introduction

Adults with cerebral palsy (CP) have a higher prevalence of many different health conditions, compared to the general population (1–4). Musculoskeletal problems such as musculoskeletal pain, contractures, and scoliosis have long been recognized as complications of CP, but recent studies have broadened the focus to also include medical and mental health. Several reports have shown that adults with CP have a higher prevalence of cardiovascular and respiratory disease, including health conditions such as heart failure, ischemic heart disease, stroke, hypertension, obesity, diabetes, and asthma, and an increased prevalence of mental health disorders such as depression and anxiety compared to the general population (1–5).

However, CP is a condition with marked variations in symptoms and severity between individuals, ranging from independent to totally dependent in all daily activities. These individual variations are often described using classification systems such as the gross motor function classification system (GMFCS) (6) and the communication function classification system (CFCS) (7). The type and localization of the neurological symptoms can be described using the CP subtype classification (8). In addition, intellectual disability is diagnosed based on standardized intelligence testing and evaluation of adaptive skills (9).

The leading cause of death in both children and adults with CP is respiratory disorders (10, 11). Both the CP subtype and the severity of impairments in childhood have been shown to be related to survival in both childhood and adulthood (10, 11). Hence, it is likely that the background of CP, the subtype and the impairments, play a role in the development of the respiratory disorders causing early mortality. These mechanisms have been well-described in children with CP (12). However, the health conditions in adults with CP might differ from those in children, as health conditions related to CP might be amalgamated with other health conditions affecting adults.

The links between the functional impairments, the co-occurring health conditions, and the causes of death in adults with CP has only recently begun to be investigated. For example, the increased prevalence of cardiovascular disease and stroke might be explained by an increased prevalence of known risk factors such as lower aerobic fitness, less muscle mass, and higher percentages of body fat. These risk factors in turn could be caused by combinations of pain, fatigue, and physical inactivity, originating from the motor impairments (13). Other links between impairments, health conditions, and causes of death in adults with CP remain to be explored, and evidence-based interventions to be established.

It is possible that early detection and treatment of several of the co-occurring health conditions could not only improve health and well-being, but also improve survival. The ideal would be for preventive measures to be tailored to the health risks of the individual, based on known characteristics, such as the CP subtype and the severity of specific impairments.

Recent studies have provided important knowledge about the prevalence of numerous health conditions in adults with CP (1–5, 14, 15). However, many of these studies are based on health care registers or claims registers that provide data on diagnostic codes, but rarely include data on CP subtype or impairment severity classifications. The association between each health condition and the CP subtype or severity of impairments in adults, is therefore largely unknown. Moreover, health conditions that are not treated, or not relevant to the reason for the consultation may not always be given a diagnostic code. Therefore, the prevalence of the various health conditions experienced by adults with CP are likely to be underreported in health care registers. Additionally, a recent review of health conditions in adults with CP, noted that individuals with intellectual disability were excluded in as many as 27% of the samples (14). Considering the increased mortality associated with intellectual disability (10, 11, 16), this should be a prioritized population to study, and excluding them is likely to affect the prevalence of the studied health conditions.

The aim of this population-based study was to look beyond the musculoskeletal aspects of CP and determine the prevalence of other common health conditions, in adults with CP in western Sweden. A second aim was to identify associations with the CP subtype, the gross motor function, the communication function, or the presence of intellectual disability.

## Materials and Methods

The present study was a population-based cross-sectional study based on interviews, physical assessments, and a manual review of medical records. The study was part of a more extensive project surveying health and participation in adults with CP in western Sweden. The method for identifying and inviting participants has been described in a previous study (17). The follow-up assessments, consisting of a thorough medical history, a physical examination, and questionnaires, were conducted during 2015–2019. Medical records were then obtained from the hospitals and habilitation units in the area. The follow-up assessments were conducted by a multiprofessional team with extensive clinical experience of patients with CP. All medical interviews were conducted by authors UJ or KH, and all medical records were reviewed by author UJ. For the purpose of facilitating the participation of individuals with intellectual disability or communication impairments, various individual adaptations, regarding for example communication methods, assistance, scheduling, and location of the assessment, were made to accommodate for the needs of each participant. In the instances where a participant was still unable to understand or answer a question, a proxy answer was recorded. All participants or their legal guardian gave informed consent. The study was approved by the Regional Ethics review board in Gothenburg 2014-01-16 No. 777-13 and the Swedish Ethical Review Authority 2019-11-05 No. 2019-05518.

### Participants

**Figure 1:**
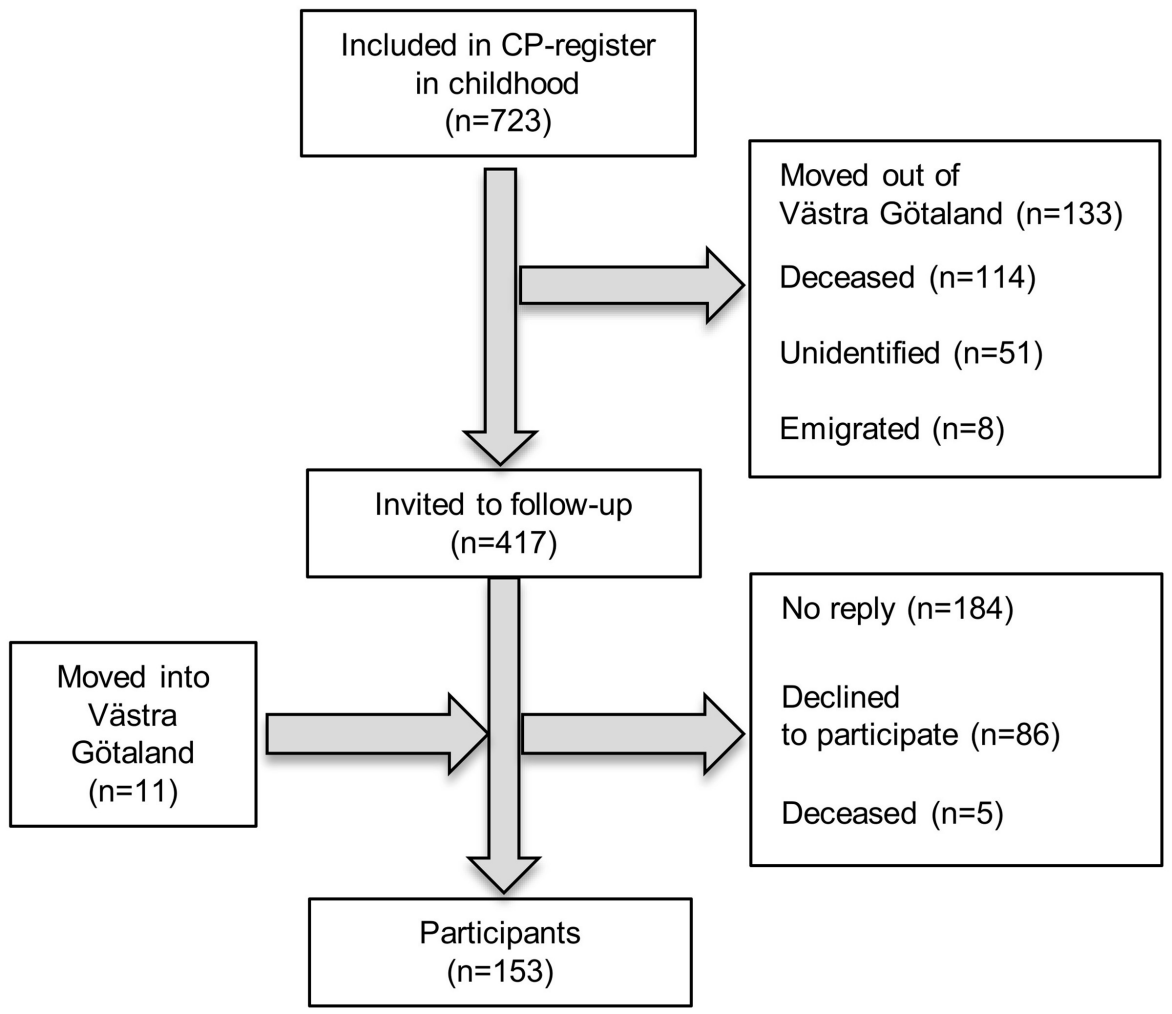
Flow-chart of the inclusion process.

All adults included in the 20 oldest birth cohorts in the CP register of western Sweden (18), born 1959–1978 and still residing in the Region of Västra Götaland (*n* = 417), were invited to a follow-up assessment. Region Västra Götaland, on the west coast of Sweden, with 1.6 million inhabitants distributed over both rural and urban areas, makes up two-thirds of the population included in the CP register of western Sweden. Adults with CP, born 1959–1978, who had moved into the area and thus were not in the register, were invited through patient organizations and habilitation units. A total of 153 adults with CP participated (Figure 1).

### Definitions

Cerebral palsy subtype was classified according to the Surveillance of CP in Europe, as unilateral spastic, bilateral spastic, dyskinetic, or ataxic CP (8). Gross motor function classification system and CFCS levels were assessed (6, 7). Intellectual disability was defined as an intellectual quotient (IQ) of <70 with deficits in adaptive skills (9). Weight (kg) and height (m) was measured and body mass index (BMI) (kg/m^2^) was calculated. Obesity was defined as having a BMI ≥30 kg/m^2^. Blood pressure was measured three times using a digital, automatic inflation device with properly sized cuffs, and a mean value was calculated. Hypertension at assessment was defined as having a mean systolic blood pressure ≥140 or diastolic blood pressure ≥90, according to the NICE guidelines (19). However, a single visit with a high blood pressure is not sufficient for a diagnosis of hypertension, and therefore only participants taking medication for hypertension were classified as having a diagnosis of hypertension.

The medical history was gathered through a semi-structured interview, covering all health concerns, both past and present. Additionally, the medical records from the children's hospital, from birth to age 18, and from adult hospitals and habilitation units from year 2000 to 2019, were reviewed. All health conditions, except pain, were classified as present if a participant self-reported having had the condition at some time, or if it was mentioned in the medical records. In order to detect all health conditions experienced by the participants, not only diagnoses and treatments were noted. Symptoms such as phlegm in airways, heartburn, or pain that were untreated or symptomatically treated without any extensive work-up, or with non-prescription medicines, were also included. Pain mentioned in medical records was often related to an acute illness, trauma, or operation and was not the persistent pain that we aimed to study. Pain was therefore defined as current or recurring pain of any type at the time of the assessment. Participants were asked to describe pain intensity, (mild, moderate, or severe), frequency (daily, weekly, monthly, or more seldom), and duration (more or less than 3 months). When participants reported several different pain sites, with different frequency, intensity, or duration, the highest intensity and frequency, and the longest duration was recorded.

Gastrointestinal tract (GI) disorders included for example: gastroesophageal reflux disease, dysphagia, gastritis, peptic ulcers, gastrostomy, constipation, diarrhea, or irritable bowel syndrome. All participants reporting either gastroesophageal reflux disease, gastritis, or peptic ulcers, or taking proton pump inhibitors, were classified as having an upper GI disorder. Psychiatric disorders were defined as any type of psychiatric disorder treated with either medication or counseling, and included for example: depression, anxiety disorders, burnout, bipolar disorder, psychotic disorders, and challenging behavior. Respiratory disorders included for example: pneumonia, asthma, sleep apnoea, and problems clearing airways of phlegm. Epilepsy was defined as having recurring seizures or taking antiepileptic medication. Diabetes included both type 1 and type 2. Pressure ulcers were defined as a pressure ulcer, grade II, or worse, according to international guidelines (20).

### Statistics

Descriptive statistics were used to document participant characteristics and the prevalence of health conditions. Age was classified in three categories: 37–44, 45–52, 53–60 years. The associations between the presence of each health condition and the age categories, the GMFCS or CFCS level were analyzed with the Mantel-Haenzsel i.e., linear-by-linear association test for trends. The associations between each health condition and sex, CP subtype, intellectual disability, or another health condition were analyzed with the Pearson Chi-square, or the Fisher's exact test when cases were few. The significance level was set at a two-tailed *p* < 0.05. All analyses were conducted using IBM SPSS Statistics version 25.

## Results

The current study comprised 153 adults with all CP subtypes and all levels of physical and intellectual disability (Table 1). The most common health problem domains were pain 65%, GI disorders 61%, psychiatric disorders 39%, and respiratory disorders 30%. The specific health conditions most commonly reported were pain 65%, upper GI disorders 33%, dysphagia 29%, epilepsy 29%, and depression 27% (Tables 2A,B).

**Table 1:** Characteristics of participants (*n* = 153).

**Age**		
median (range)	48 years 3 months	(37–58 years)
IQR		42–55 years
**Age categories**		
37–44 years	50	(33)
45–52 years	52	(34)
53–60 years	51	(33)
**Sex**		
Female	66	(43)
Male	87	(57)
**CP subtype**		
Unilateral spastic	63	(41)
Bilateral spastic	55	(36)
Dyskinetic	29	(19)
Ataxic	6	(4)
**GMFCS level**		
I	60	(39)
II	32	(21)
III	18	(12)
IV	26	(17)
V	17	(11)
**CFCS level**		
I	109	(71)
II	16	(11)
III	10	(7)
IV	10	(6)
V	8	(5)
**Intellectual disability**		
IQ <70	34	(22)
IQ > 70	119	(78)

*Values are n (%). CP, cerebral palsy; GMFCS, gross motor function classification system; CFCS, communication function classification system; IQ, intelligence quotient*.

**Table 2A:** Health conditions in adults with CP, by CP subtype, GMFCS level, CFCS level, and ID (*n* = 153).

	**Total**		**Pain**		**Gastrointestinal**								**Psychiatric**					
					**GI total**		**Upper GI**		**Dysphagia**		**Constipation**		**Psychiatric total**		**Depression**		**Anxiety**	
Total	153	(100)	100	(65)	94	(61)	50	(33)	45	(29)	39	(26)	60	(39)	41	(27)	18	(12)
CP subtype			*p* = 0.029^*^		*p* < 0.001^*^		*p* < 0.001^*^		*p* < 0.001^*^		*p* < 0.001^*^		*p* = 0.378		*p* = 0.066		*p* = 0.021^*^	
Unilateral	63	(41)	36	(57)	19	(30)	6	(10)	8	(13)	2	(3)	20	(32)	15	(24)	3	(5)
Bilateral	55	(36)	43	(78)	43	(78)	27	(49)	20	(36)	14	(26)	24	(44)	13	(24)	12	(22)
Dyskinetic	29	(19)	19	(66)	26	(90)	16	(55)	16	(55)	18	(62)	14	(48)	13	(45)	2	(7)
Ataxic	6	(4)	2	(33)	6	(100)	1	(17)	1	(17)	5	(83)	2	(33)	0	(0)	1	(17)
GMFCS level			*p* = 0.407		*p* < 0.001^*^		*p* < 0.001^*^		*p* < 0.001^*^		*p* < 0.001^*^		*p* = 0.488		*p* = 0.142		*p* = 0.931	
I	60	(39)	37	(62)	20	(33)	9	(15)	5	(8)	2	(3)	21	(35)	14	(23)	6	(10)
II	32	(21)	21	(66)	21	(66)	10	(31)	8	(25)	7	(22)	12	(38)	6	(19)	4	(13)
III	18	(12)	12	(67)	12	(67)	5	(28)	6	(33)	5	(28)	9	(50)	5	(28)	3	(17)
IV	26	(17)	18	(69)	24	(92)	16	(62)	12	(46)	11	(42)	12	(46)	11	(42)	4	(15)
V	17	(11)	12	(71)	17	(100)	10	(59)	14	(82)	14	(82)	6	(17)	5	(29)	1	(6)
CFCS level			*p* = 0.021^*^		*p* < 0.001^*^		*p* = 0.006^*^		*p* < 0.001^*^		*p* < 0.001^*^		*p* = 0.092		*p* = 0.282		*p* = 0.067	
I	109	(71)	76	(70)	53	(49)	27	(25)	17	(16)	12	(11)	45	(41)	28	(26)	15	(14)
II	16	(11)	11	(69)	14	(88)	9	(56)	7	(44)	8	(50)	9	(56)	9	(56)	3	(19)
III	10	(7)	5	(50)	9	(90)	5	(50)	7	(70)	5	(50)	2	(20)	2	(20)	0	(0)
IV	10	(6)	5	(50)	10	(100)	4	(40)	9	(90)	6	(60)	3	(30)	2	(20)	0	(0)
V	8	(5)	3	(38)	8	(100)	5	(63)	5	(63)	8	(100)	1	(13)	0	(0)	0	(0)
ID			*p* = 0.004^*^		*p* < 0.001^*^		*p* < 0.061		*p* < 0.001^*^		*p* < 0.001^*^		*p* = 0.692		*p* = 0.826		*p* = 0.365	
IQ <70	34	(22)	15	(44)	32	(94)	16	(47)	21	(62)	23	(68)	12	(35)	8	(24)	2	(6)
IQ>70	119	(78)	85	(71)	62	(52)	34	(29)	24	(20)	16	(13)	48	(40)	33	(28)	16	(13)

*Values are n (%)*.

* *Significant, p < 0.05. p-values refer to Fisher's exact test, for variation across subtypes or presence of ID and to Linear-by-linear Association across GMFCS or CFCS-levels. Percentages are the proportion of participants with the given subtype of CP, GMFCS level, CFCS level or ID, with a certain health condition. Missing data for hypertension at visit (n = 7). CP, cerebral palsy; GMFCS, gross motor function classification system; CFCS, communication function classification system; ID, intellectual disability; IQ, intelligence quotient; pain, recurring or consistent pain; GI, gastrointestinal tract; GI total, includes upper GI, dysphagia, constipation, and miscellaneous GI disorders; upper GI, gastroesophageal reflux and gastritis, psychiatric total includes depression, anxiety and miscellaneous psychiatric disorders*.

**Table 2B:** Health conditions in adults with CP, by CP subtype, GMFCS level, CFCS level, and ID (*n* = 153).

	**Respiratory**								**Other**									
	**Respiratory total**		**Phlegmy**		**Pneumonia**		**Asthma**		**Hypertension at visit**		**Hypertension diagnosis**		**Diabetes**		**Epilepsy**		**Pressure ulcers**	
Total	46	(30)	19	(12)	15	(10)	9	(6)	63	(41)	27	(18)	12	(8)	44	(29)	14	(9)
CP subtype	*p* = 0.006^*^		*p* < 0.001^*^		*p* = 0.023^*^		*p* = 0.354		*p* = 0.240		*p* = 0.245		*p* = 0.838		*p* = 0.122		*p* = 0.048^*^	
Unilateral	10	(16)	0	(0)	4	(6)	2	(3)	24	(39)	9	(14)	4	(6)	23	(37)	2	(3)
Bilateral	20	(36)	9	(16)	3	(6)	6	(11)	28	(54)	13	(24)	6	(11)	11	(20)	6	(11)
Dyskinetic	14	(48)	9	(31)	6	(21)	1	(3)	10	(37)	3	(10)	2	(7)	7	(24)	6	(21)
Ataxic	2	(33)	1	(17)	2	(33)	0	(0)	1	(20)	2	(33)	0	(0)	3	(50)	0	(0)
GMFCS level	*p* < 0.001^*^		*p* < 0.001^*^		*p* = 0.013^*^		*p* = 0.907		*p* = 0.133		*p* = 0.972		*p* = 0.918		*p* = 1.000		*p* < 0.001^*^	
I	8	(13)	1	(2)	3	(5)	2	(3)	33	(55)	8	(13)	4	(7)	17	(28)	1	(2)
II	9	(28)	2	(6)	2	(6)	5	(16)	8	(26)	8	(25)	4	(13)	10	(31)	0	(0)
III	6	(33)	1	(6)	3	(17)	0	(0)	8	(47)	4	(22)	1	(6)	6	(33)	1	(6)
IV	13	(50)	9	(35)	2	(8)	1	(4)	10	(40)	6	(23)	2	(8)	3	(12)	5	(19)
V	10	(59)	6	(35)	5	(29)	1	(6)	4	(31)	1	(6)	1	(6)	8	(47)	7	(41)
CFCS level	*p* = 0.030^*^		*p* = 0.003^*^		*p* < 0.001^*^		*p* = 0.199		*p* = 0.043^*^		*p* = 0.028^*^		*p* = 0.715		*p* < 0.001^*^		*p* < 0.001^*^	
I	26	(24)	7	(6)	5	(5)	8	(7)	52	(48)	24	(22)	9	(8)	23	(21)	5	(5)
II	8	(50)	4	(25)	3	(19)	1	(6)	4	(27)	2	(13)	1	(6)	4	(27)	0	(0)
III	4	(40)	3	(30)	2	(20)	0	(0)	5	(56)	0	(0)	1	(10)	5	(25)	3	(30)
IV	4	(40)	3	(30)	2	(20)	0	(0)	2	(22)	1	(10)	1	(10)	6	(60)	3	(30)
V	4	(50)	2	(25)	3	(38)	0	(0)	0	(0)	0	(0)	0	(0)	6	(75)	3	(38)
ID	*p* = 0.056		*p* = 0.015^*^		*p* = 0.001^*^		*p* = 0.685		*p* < 0.035^*^		*p* = 0.043^*^		*p* = 0.302		*p* = 0.001^*^		*p* < 0.001^*^	
IQ <70	15	(44)	9	(27)	9	(27)	1	(3)	7	(25)	2	(6)	1	(3)	18	(53)	12	(35)
IQ>70	31	(26)	10	(8)	6	(5)	8	(7)	56	(48)	25	(21)	11	(9)	26	(22)	2	(2)

*Values are n (%)*.

* *= significant, p < 0.05. p-values refer to Fisher's exact test, for variation across subtypes or presence of ID and to Linear-by-linear Association across GMFCS or CFCS-levels. Percentages are the proportion of participants with the given subtype of CP, GMFCS level or ID, with a certain health condition. Missing data for hypertension at visit (n = 7). CP, cerebral palsy; GMFCS, gross motor function classification system; CFCS, communication function classification system; ID, intellectual disability; IQ, intelligence quotient; Respiratory total includes Phlegmy, pneumonia, asthma and miscellaneous respiratory disorders; Hypertension at visit ≥140/≥90 mmHg; hypertension diagnosis, treated for hypertension; diabetes, type 1 and 2*.

Among the 100 participants reporting pain, 58% reported moderate pain, 47% daily pain, and 75% a pain duration of over 3 months, while only 30% reported taking pain medication (Table 3). Pain was significantly associated with CP subtype, and was less often reported from participants with a more impaired communication (a less functional CFCS level) or with intellectual disability (Table 2). There was no association between pain and GMFCS level in the total group. However, subgroup analysis showed that among participants without intellectual disability pain was more common in participants with a less functional GMFCS level (*p* = 0.022). Among participants with intellectual disability there was no association between pain and GMFCS level.

**Table 3:** Pain characteristics (*n* = 100).

**Intensity**		
Mild	17	(17)
Moderate	58	(58)
Severe	25	(25)
**Frequency**		
Monthly	13	(13)
Weekly	23	(23)
Daily	47	(47)
Unknown	17	(17)
**Duration**		
<3 months	8	(8)
>3 months	75	(75)
Unknown	17	(17)
**Pain medication**		
Yes	30	(30)

*Values are n (%)*.

The GI disorders most commonly reported were upper GI disorders, dysphagia, and constipation (Table 2). Dysphagia was associated with pneumonia (*p* = 0.041) and problems with phlegm (*p* = 0.006). Five participants had a gastrostomy and all five had an intellectual disability and were classified as GMFCS and CFCS levels IV or V.

The most common psychiatric disorder was depression, and the most common respiratory disorders were pneumonia and problems with phlegm (Table 2). Twelve participants were treated with inhalations for airway symptoms such as asthma or phlegm. Chronic obstructive pulmonary disease (COPD) or chronic bronchitis was not reported by any participant nor in any medical records.

While 18% were treated for hypertension, 41% had a blood pressure of ≥140/90 mmHg measured at the visit (Table 2). The median BMI was 25.9 kg/m^2^ and the median waist circumference 94 cm. Underweight was significantly related to male sex, a less functional GMFCS and CFCS level, and intellectual disability (Table 4). All six participants who were underweight had both intellectual disability and dysphagia but only two had a gastrostomy.

**Table 4:** Waist and body mass index (BMI) in adults with CP, by age, sex, CP subtype, and impairments (*n* = 152).

	**Waist**		**BMI**		**Underweight**			**Obesity**		
All	94		25.9		6	(4)		32	(21)	
Age		*p* = 0.606^a^		*p* = 0.942^a^			*p* = 0.452^c^			*p* = 0.809^c^
37–44 years	92		26.2		2	(4)		12	(24)	
45–52 years	92		25.9		0	(0)		9	(18)	
53–60 years	94		25.9		4	(8)		11	(22)	
Sex		*p* = 0.248^a^		*p* = 0.413^a^			*p* = 0.036^*^^b^			*p* = 1.000^b^
Female	92		26.5		0	(0)		14	(21)	
Male	95		25.6		6	(7)		18	(21)	
CP subtype		*p* = 0.019^*^^a^		*p* = 0.003^*^^a^			*p* = 0.250^b^			*p* = 0.084^b^
Unilateral	95		27.0		1	(2)		16	(25)	
Bilateral	95		26.4		2	(4)		14	(26)	
Dyskinetic	88		23.6		3	(10)		2	(7)	
Ataxic	85		24.5		0	(0)		0	(0)	
GMFCS level		*p* = 0.585^a^		*p* = 0.297^a^			*p* = 0.001^*^^c^			*p* = 1.000^c^
I	94		26.3		0	(0)		13	(22)	
II	94		26.1		1	(3)		7	(22)	
III	93		26.2		0	(0)		3	(17)	
IV	93		25.5		1	(4)		5	(20)	
V	81		20.8		4	(24)		4	(24)	
CFCS level		*p* = 0.056^a^		*p* = 0.050^*^^a^			*p* < 0.001^*^^c^			*p* = 0.090^c^
I	95		26.4		0	(0)		28	(26)	
II	90		24.9		1	(6)		1	(6)	
III	89		22.7		0	(0)		1	(11)	
IV	81		20.1		3	(30)		1	(10)	
V	77		20.5		2	(25)		1	(13)	
ID		*p* = 0.073^a^		*p* = 0.330^a^			*p* < 0.001^*^^b^			*p* = 0.642^b^
IQ < 70	85		22.9		6	(18)		6	(18)	
IQ > 70	94.0		26		0	(0)		26	(22)	

*Values for Wast and BMI are median, values for underweight and obesity are n (%)*,

* *Significant, p < 0.05. p-values are*

a *Median test*,

b *Fisher's exact test*,

c *Mantel-Haenzsel Linear-by-linear Association. Missing data for waist (n = 2). CP, cerebral palsy; GMFCS, gross motor function classification system; CFCS, communication function classification system; ID, intellectual disability; IQ, intelligence quotient; Waist, waist circumference in cm; BMI, body mass index, underweight, BMI <18.5 kg/m^2^; Obesity, BMI ≥ 30 kg/m^2^*.

The presence of health conditions varied with the nature and severity of impairments. For example, participants with dyskinetic CP were the most affected by both upper GI disorders, dysphagia, and respiratory disorders, and a less functional GMCS level was significantly associated with a higher prevalence of GI disorders, respiratory disorders, and pressure ulcers. Intellectual disability was significantly associated with several health conditions, but also with a lower prevalence of pain and hypertension (Table 2).

There were no significant associations between age or sex and CP subtypes, impairments, or health conditions, except for an association between sex and high blood pressure measured at the visit (Table 5 in Supplementary Material).

The CFCS level was very closely related to the presence of intellectual disability (*p* < 0.001), with 94% of participants without intellectual disability classified as CFCS level 1.

## Discussion

The most common health conditions in this population-based study of adults with CP of all CP subtypes and levels of physical and intellectual impairment, were pain, upper GI disorders, dysphagia, epilepsy, and depression. Additionally, the prevalence of several common health conditions were shown to be significantly related to the CP subtype, the GMFCS level, the CFCS level, or the presence of intellectual disability.

The high prevalence of pain in our study was in accordance with a recent systematic review of pain in adults with CP (15). In our study pain was less frequently reported by participants with intellectual disability or a more impaired communication, in spite of the adaptations made to enable all participants to answer for themselves. A possible explanation for this could be that some individuals with intellectual disability might not be able to understand or express that the discomfort they feel is pain. In addition, some might express pain non-verbally in ways that their carers don't always recognize and others may be so accustomed to a chronic pain that they don't express it at all. Many adults in our study reported pain but took no medication. This was maybe not so surprising, as chronic pain is often better managed with other strategies than pain medication. Many also reported avoiding activities in daily life that they knew would cause pain. According to earlier studies, adults with CP don't always access health care providers for help with managing pain, and a majority of those who receive treatments still have pain (21, 22). The pain in CP can have a great variety of origins, including musculoskeletal, neurogenic, or internal organs, and assessment can be challenging (21, 23). Thus, there is a risk that adults with CP are missing out on suitable interventions for alleviating pain and there is also the possibility that some adults with CP have pain with an origin that could have been prevented, treated, or cured instead of coped with.

A majority of our participants reported GI disorders. Though dysphagia, gastroesophageal reflux disease, and chronic constipation are frequent problems in the clinic, and the mechanisms by which CP causes GI disorders has been well-described in children with CP (24), there is a paucity of research on adults with CP, both on how these mechanisms affect adults and on how they best can be treated (25, 26).

The proportion of participants who reported that they had been treated for psychiatric disorders such as depression and anxiety, at some point in their lives, was higher than in previous studies of adults with CP (1, 4). This is what could be expected, since our study covered a longer time period and included treatments other than medication. Interestingly, in our study the prevalence of depression and anxiety were not related to any of the other impairments studied.

The prevalence of epilepsy was high compared to previous studies (14). There are several possible explanations for this. Firstly, the prevalence of epilepsy is closely related to the prevalence of intellectual disability in the study sample. Secondly, in our study both resolved and current epilepsy, from birth to middle age, was included. We have previously shown that epilepsy in CP indeed can be resolved (17), but nevertheless it remains one of the most common and consequential health conditions in CP.

One-third of our participants reported respiratory disorders, but only 6% reported having asthma and no participants reported COPD. Some cases of pneumonia were classified as aspiration pneumonia in the medical records, but in many cases the cause of the pneumonia could not be ascertained. Respiratory disorders is a complex issue in adults with CP, with spasticity and weakness, gastroesophageal reflux disease and dysphagia causing chronic aspiration, chronic airway inflammation, poor airway clearance, and impaired lung function, all of which then predispose for respiratory infections and respiratory failure (12, 27). However, in health care registers, the respiratory disorder most often found in adults with CP is asthma (1, 3). It seems possible that the diagnosis of asthma is used as a sort of proxy, for lack of better diagnostic codes, but it might also be a sign of health care professionals lacking insight into the complexity of respiratory disorders in adults with CP. According to our results, respiratory disorders such as pneumonia and problems with phlegm, were more common in participants with dysphagia than in those without, and more common in participants with a dyskinetic CP subtype, a less functional GMFCS level, or intellectual disability. This finding provides a link between the studies showing shorter survival in individuals with any of these impairments, and the studies showing respiratory disorders to be the leading cause of death in adults with CP. However, there is currently little or no evidence for the interventions used for prevention and management of respiratory disorders in individuals with CP, making this an important area for future research (28).

High blood pressure measured at the visit was twice as common (41%) compared to the number of participants who had a diagnosis of hypertension and were using antihypertensive medication (18%). In comparison, a recent systematic review of hypertension in CP reported a 28.9% prevalence of hypertension and 0–18% using antihypertensive medication (29). They found no difference related to GMFCS levels, but the association with intellectual disability seems not to have been analyzed. Surprisingly, our results show a markedly lower prevalence of hypertension among participants with intellectual disability. This finding is contrary to recent studies of hypertension in adults without CP, stating a prevalence of 31.1% in all adults (30), and 36.7% in adults with intellectual disability (31) and at this stage we have found no explanation for it. The high prevalence of high blood pressure measured at the visit might partly be due to the “white coat effect,” since the visit was a potentially stressful situation for the participant (19). Of course, there could also be hitherto undetected cases of hypertension. Since a single visit with a high blood pressure is not sufficient for a diagnosis of hypertension, all participants with high blood pressure at the visit were recommended to contact a general practitioner (GP) for a proper assessment.

The median BMI and the prevalence of obesity were in line with a recent systematic review (14), whereas the median waist circumference was higher. In adults with CP, where the motor impairment might cause lower muscle mass and bone density, waist circumference might be a better indicator of excessive body fat and cardiovascular risk (13). The prevalence of obesity was not related to age, sex, or any of the impairments. In contrast, underweight was related to male sex, a less functional GMFCS and CFCS level and to intellectual disability. Similar associations were observed for pressure ulcers, underscoring the medical vulnerability of the most severely impaired adults with CP.

According to our results, intellectual disability was associated with an increased prevalence of a variety of health conditions, most notably epilepsy, GI disorders, pneumonia, and pressure ulcers. To our knowledge this has not been studied in adults with CP before. Even though the individuals who are still alive are likely to have been the healthiest, these findings provide important information regarding the factors behind the increased risk of early mortality in adults in this population. To some extent, these health conditions and causes of death might be preventable. Adults with intellectual disability constitute a high risk population, many of whom have difficulties identifying symptoms or are dependent on others for seeking medical attention, and who therefore should be offered regular health checks (32, 33). Our results confirm the importance of providing regular follow-up and preventive care for adults with CP and intellectual disability.

The associations between CFCS levels and health conditions were affected by the close relationship between CFCS levels and intellectual disability, and the individuals with intellectual disability were too few for meaningful subgroup analyses. Nonetheless, it is likely that examining a larger population of individuals with intellectual disability would reveal associations between health conditions and communication function. Not only in accessing primary care, but also during hospital care, individuals with communication impairments can have difficulties gaining the attention of the medical staff and communicating their symptoms, and for this reason have an increased risk of complications and death (11, 34, 35).

The finding that the prevalence of several health conditions were related to the CP subtype and the severity of impairments indicates that CP might be a factor in the development of these health conditions. If CP is involved in the development of a health condition, it seems reasonable that it might also affect the effectiveness of treatments. For example, there are other mechanisms leading to recurring respiratory infections in CP than in the general population, and treatments need to be chosen with this in mind (27). Hence, both preventive health care and standard treatments used for the general population might need to be adapted to suit the specific health risks, increased morbidity, and early mortality of adults with CP.

Further research is needed to clarify how symptoms, disease mechanisms, and treatments for specific health conditions might differ between adults with CP and the general population.

### Limitations

A major strength of this study is the use of a combination of a population-based CP-register for the identification of adults with CP, and a follow-up visit during which current impairments were assessed and self-reported health conditions were recorded. At the same time, the interview and assessment demanded considerable time and effort from the participants and their carers, maybe leading to a low response rate.

The medical records from hospitals and habilitation units were reviewed. However, many health conditions, such as pain, phlegm in the airways, dyspepsia, or constipation, had been treated mainly in primary care. Even though these health conditions were often mentioned in the medical history in hospital or habilitation records, they were seldom the reason for the visit and were therefore not worked-up or coded for. If the participants did not remember them during our interview, such health conditions could be underreported in our results. In particular, adults with intellectual disability living in group home facilities and being cared for by GPs might be less likely to be referred to hospital care and less likely to report all experienced health conditions. In addition, health conditions might be underreported because of the difficulties adults with CP experience in accessing care, and of diagnostic overshadowing, where new symptoms are attributed to CP instead of a comorbid condition.

The health conditions presented in this study were self-reported or as mentioned in medical records. The prevalence of each health condition needs to be interpreted with this in mind. Future work is required to further investigate and objectively verify their prevalence. It seems likely, however, that the associations described between CP subtypes, impairments and health conditions would be relevant to objectively verified, as well as subjectively described health conditions.

Some childhood data on the non-responders were available from the register. There were no differences regarding sex, age, or GMFCS level, but the prevalence of intellectual disability and epilepsy was higher in non-responders (Table 6 in Supplementary Material). Since participants with ID had a higher prevalence of many health conditions, the true prevalence of these health conditions in the population of adults with CP is likely to be somewhat higher than in our results. Even so, this should not affect the associations between the CP subtypes, impairments, and health conditions.

### Conclusion

Adults with CP have health conditions stemming from several different organ systems. Hence, follow-up and preventive care for adults with CP needs to include not only the impairments, but an assessment of general health as well. Knowledge of how the prevalence of specific health conditions is related to CP subtype and impairment severity can help individualize preventive care and also inspire future research into disease mechanisms and treatments.

## Data Availability Statement

The datasets presented in this article are not readily available because of privacy and ethical restrictions. The anonymized data that support the findings of this study are available on request from the corresponding author. Requests to access the datasets should be directed to ulrica.jonsson@neuro.gu.se.

## Ethics Statement

The studies involving human participants were reviewed and approved by the Swedish Ethical Review Authority. The participants or their legal guardians provided their written informed consent to participate in this study.

## Author Contributions

UJ and KH conceptualized and designed the study and analyzed and interpreted the data. UJ, KH, and ME were involved in the data collection. UJ drafted the manuscript. All authors critically revised the manuscript and approved the final manuscript.

## Funding

The study has been supported by Grants from the Swedish state under the agreement of the Swedish government and the county councils, the ALF agreement SU 2019-02662 and SU 2018-04276, the Health & Medical Care Committee of the Region Västra Götaland, the Region Västra Götaland, Habilitation & Health, the Sahlgrenska University Hospital Foundations, the NEURO Sweden, and the Norrbacka-Eugenia Foundation. No funding body had any role in the design, the collection, and interpretation of data or the writing of the manuscript.

## Conflict of Interest

The authors declare that the research was conducted in the absence of any commercial or financial relationships that could be construed as a potential conflict of interest.

## Publisher's Note

All claims expressed in this article are solely those of the authors and do not necessarily represent those of their affiliated organizations, or those of the publisher, the editors and the reviewers. Any product that may be evaluated in this article, or claim that may be made by its manufacturer, is not guaranteed or endorsed by the publisher.
