# Supplementary material for: Health Conditions in Adults With Cerebral Palsy: The Association With CP Subtype and Severity of Impairments
Source: Front Neurol. 2021 Oct 28;12:732939. doi: 10.3389/fneur.2021.732939 (PMC8581638; doi:10.3389/fneur.2021.732939)
Supplement: Supplementary file 1 [file Table_5.pdf]

Table 5. Impairments and health conditions by sex and age (n=153).

	Sex			Age			
	Female	Male		37-44y	45-52y	53-58y	
Total	66	87		50	52	51	
CP subtype			0.394 [a]				0.876 [a]
Unilateral spastic	23	40		19	23	21	
Bilateral spastic	25	30		18	19	18	
Dyskinetic	14	15		11	7	11	
Ataxic	4	2		2	3	1	
GMFCS level			0.772 [a]				0.144 [b]
I	23	37		17	26	17	
II	14	18		6	7	19	
III	10	8		9	5	4	
IV	11	15		11	9	6	
V	8	9		7	5	5	
CFCS level			0.684 [a]				0.900 [b]
I	46	63		34	39	36	
II	8	8		6	3	7	
III	3	7		4	4	2	
IV	4	6		4	4	2	
V	5	3		2	2	4	
Intellectual disability			0.239 [a]				0.448 [a]
IQ<70	18	16		14	9	11	
IQ>70	48	71		36	43	40	
Health conditions							
Pain	45	55	0.608 [a]	33	32	35	0.835 [b]
GI total	43	51	0.503 [a]	31	33	30	0.761 [b]
Upper GI	18	32	0.229 [a]	19	18	13	0.205 [b]
Dysphagia	25	20	0.051 [a]	20	11	14	0.192 [b]
Constipation	18	21	0.710 [a]	16	11	12	0.364 [b]
Psychiatric total	31	29	0.097 [a]	22	16	22	1.000 [b]
Depression	23	18	0.065 [a]	18	8	15	0.503 [b]
Anxiety	8	10	1.000 [a]	5	6	7	0.646 [b]
Respiratory total	21	25	0.724 [a]	16	14	16	1.000 [b]
Phlegmy	11	8	0.217 [a]	5	7	7	0.654 [b]
Pneumonia	5	10	0.585 [a]	3	4	8	0.133 [b]
Asthma	4	5	1.000 [a]	3	3	3	1.000 [b]
Hypertension at visit	21	42	0.044* [a]	20	22	21	0.837 [b]
Hypertension diagnosis	9	18	0.291 [a]	7	7	13	0.153 [b]
Diabetes	4	8	0.555 [a]	2	3	7	0.096 [b]
Epilepsy	17	27	0.589 [a]	14	16	14	1.000 [b]
Pressure ulcers	8	6	0.397 [a]	6	4	4	0.498 [b]

All values are n. * significance level $p < 0.05$. p-values are [a]Fisher´s exact test or [b] Mantel-Hanzel Linearby-linear Association for trends. GMFCS, Gross Motor Function Classification System; CFCS, Communication Function Classification System; IQ, Intelligence Quotient; GI, Gastrointestinal disorders.
