# Supplementary material for: Health Conditions in Adults With Cerebral Palsy: The Association With CP Subtype and Severity of Impairments
Source: Front Neurol. 2021 Oct 28;12:732939. doi: 10.3389/fneur.2021.732939 (PMC8581638; doi:10.3389/fneur.2021.732939)
Supplement: Supplementary file 2 [file Table_6.pdf]

Table 6. CP-register data for participants and non-responders.

	Participants n=142	Participants vs non-responders	Non-responders n=275
Sex		p=0.898[a]	
female	60(42)		118(43)
male	82(58)		157(57)
CP subtype		*p=0.001[a]	
unilateral	62(44)		101(37)
bilateral	50(35)		132(48)
dyskinetic	24(17)		19(7)
ataxic	6(4)		23(8)
Walking impairment		p=0.126[b]	
mild (GMFCS I-II)	102(71)		186(68)
moderate (GMFCS III)	15(11)		35(13)
severe (GMFCS IV-V)	25(18)		45(16)
unknown	0		9(3)
Intellectual disability	23(16)	*p=0.004[a]	80(29)
Epilepsy	13(9)	*p=0.006[a]	54(19)

Data are n(%). CP, Cerebral Palsy; GMFCS, Gross Motor Function Classification System. a=Pearson chi-square, b=Fisher´s exact test
*=significant difference p<0.05
